# An oral lichen planus–like mouse model driven by IFN-**γ** signaling and cytotoxic CD8^+^ T cells

**DOI:** 10.1172/jci.insight.185380

**Published:** 2025-12-11

**Authors:** Zhenlai Zhu, Tinglan Yang, Peng Peng, Kang Li, Wen Qin, Chen Zhang, Shuyan Wang, Yuanyuan Wang, Minghui Wei, Erle Dang, Meng Fu, Hao Guo, Wen Yin, Shuai Shao, Qing Liu

**Affiliations:** 1 State Key Laboratory of Oral & Maxillofacial Reconstruction and Regeneration, National Clinical Research Center for Oral Diseases, Shaanxi Clinical Research Center for Oral Disease, Department of Oral medicine, School of Stomatology,; 2 Department of Dermatology, Xijing Hospital,; 3 Department of Microbiology & Pathogen Biology, School of Basic Medical Sciences,; 4 Department of Transfusion Medicine, Xijing Hospital, and; 5 State Key Laboratory of Oral & Maxillofacial Reconstruction and Regeneration, National Clinical Research Center for Oral Diseases, Shaanxi Clinical Research Center for Oral Diseases, Department of Preventive Dentistry, School of Stomatology, Fourth Military Medical University, Xi’an, Shaanxi, China.

**Keywords:** Dermatology, Immunology, Inflammation, Mouse models, Skin, T cells

## Abstract

Oral lichen planus (OLP) is a recalcitrant inflammatory disease with potential for malignant transformation, involving a cytotoxic CD8^+^ T cell–mediated basal keratinocyte apoptosis. However, it lacks an appropriate mouse model for study. Here we developed an OLP-like mouse model using topical oxazolone to induce a delayed-type hypersensitivity-mediated oral lichenoid reaction. Histological and ultrastructural analysis confirmed hallmark pathological features of OLP, including band-like CD8^+^ T cell infiltration and basal cell damage as well as the presence of Civatte bodies. Comparative transcriptomic analysis revealed significant similarity between RNA-Seq profiles of the mouse model and human OLP lesions, highlighting shared upregulated genes and enriched pathways, particularly those related to IFN-γ signaling and cytotoxic T cell activity. Functional studies demonstrated that the OLP phenotype depended on IFN-γ, with local priming by IFN-γ intensifying the disease through upregulation of major histocompatibility complex class I. Additionally, the absence of Langerhans cells exacerbated disease severity in vivo. Therapeutic evaluation showed that the JAK inhibitors baricitinib and ruxolitinib effectively reduced disease burden and provided mechanistic insights. In conclusion, this OLP-like mouse model recapitulates key immunopathological and transcriptomic features of human OLP, offering a robust platform for dissecting disease mechanisms and evaluating novel therapeutic strategies.

## Abstract 2

A novel mouse model replicates oral lichen planus, facilitating the study of disease mechanisms and potential therapeutic strategies.

## Graphical abstract

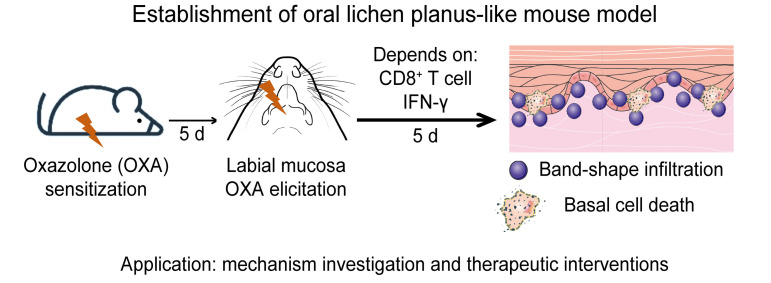

## Introduction

Oral lichen planus (OLP) is a chronic, persistent inflammatory disorder that manifests as reticular, erythematous, or ulcerative lesions in the oral mucosa (1). It carries an increased risk of progression to oral squamous cell carcinoma (2, 3). While the precise etiology remains unclear, OLP pathogenesis is driven by reactive CD8^+^ T cells targeting unknown antigens presented by dendritic cells (4). This process leads to CD8^+^ T cell activation, IFN-γ production, and apoptosis of basal keratinocytes (4, 5). Shared immunological mechanisms underlie the different clinical variants of lichen planus, though their manifestations and natural histories vary (6, 7). Our previous research demonstrated that IFN-γ is a key mediator in lichen planus progression, upregulating major histocompatibility complex class I (MHC I) expression via the JAK/STAT pathway (8). Treatment options are limited primarily to off-label use of topical steroids (2, 9). Recently, JAK inhibitors have shown promise as potential therapeutic agents (10), but their efficacy and safety require further investigation.

Animal models are essential for understanding the mechanisms underlying OLP and for testing new therapies. In contrast to other inflammatory conditions such as psoriasis (11) or atopic dermatitis (12), where robust animal models are available, models that accurately replicate OLP are scarce (13–17). Existing approaches, including xenogeneic T cell transfer, exposure to amalgam, or *E*. *coli* infection under zinc deficiency, have been used to induce oral lichenoid reactions (OLRs) with some OLP-like features. However, inconsistencies in lesion development and complex modeling processes have limited their utility. Thus, there is an urgent need for a simple, practical, and reproducible mouse model that closely mimics human OLP. OLRs, which closely resemble OLP clinically and histologically, share similarities in their inflammatory processes, characterized by lymphocytic infiltration and basal cell damage (18–20). Both conditions involve antigen sensitization, clonal expansion of lymphocytes, and inflammatory responses triggered by antigen reexposure (18, 21). CD8^+^ T cells and IFN-γ are recognized as pivotal players in the inflammatory cascade of OLP (5, 22, 23). The key distinction lies in etiology and reversibility: OLP is a chronic, Th1-skewed immune-mediated disease of unknown antigenic specificity, whereas OLR arises from identifiable external triggers and typically resolves upon their removal (18, 19, 21). Type IV hypersensitivity consists of a sensitization phase and an elicitation phase, primarily mediated by cytotoxic CD8^+^ T cells and IFN-γ. Therefore, using sensitizing agents to establish type IV hypersensitivity–mediated OLR can be a feasible method to develop an OLP-like mouse model.

In this study, we aimed to establish a reliable OLP-like mouse model using the hapten oxazolone (OXA). Histological analysis revealed hallmark OLP-like characteristics, including CD8^+^ T cell infiltration, basal cell liquefaction, colloid body, and keratinocyte apoptosis. Transcriptomic profiling further confirmed similarities with patient-derived OLP lesions. Functional studies highlighted the critical roles of CD8^+^ T cells and IFN-γ, with IFN-γ priming exacerbating disease severity. Importantly, treatment with the JAK inhibitors baricitinib (oral) and ruxolitinib (topical) significantly ameliorated OLP-like phenotype. Together, these findings demonstrate that our modified mouse model provides a robust platform for studying OLP pathogenesis and testing therapeutic strategies.

## Results

### OLP-like mouse model is established by topical OXA application.

**Figure 1:**
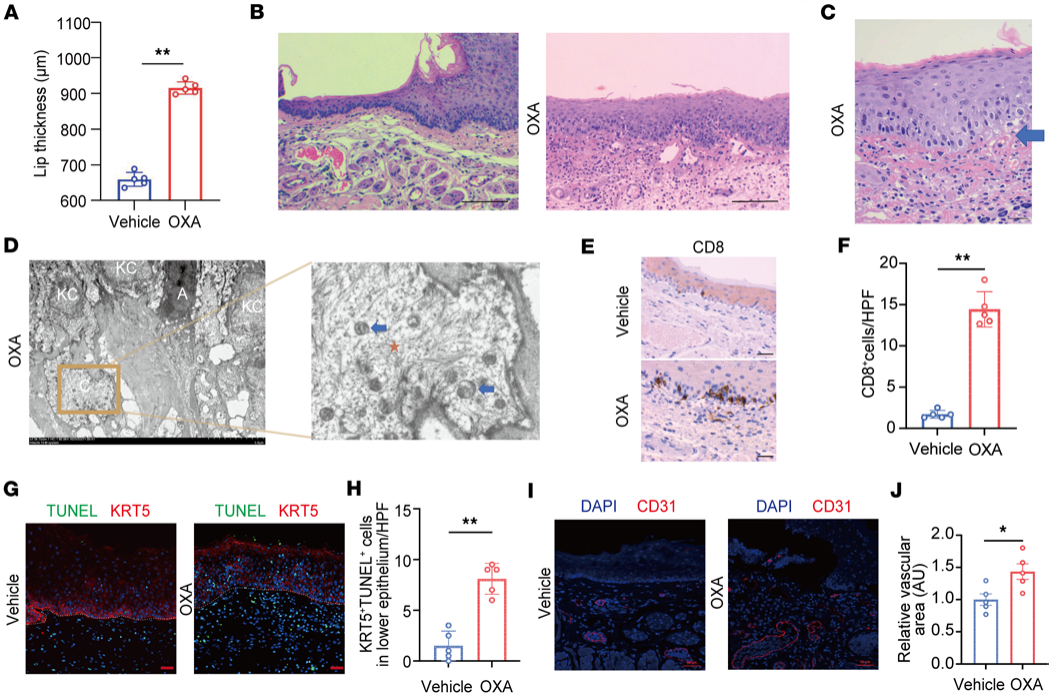
Establishment of an OLP-like mouse model. (**A**) Measurement of lip thickness on the fifth day in mice treated with vehicle or OXA. (**B**) Representative H&E-stained sections showing band-like lymphocytic infiltration around the basal membrane in OXA-treated mice (original magnification, ×100; scale bar: 100 μm). (**C**) Representative Civatte body in OLP-like lesions after OXA application (original magnification, ×400; scale bar: 25 μm). Blue arrow indicates Civatte body near the base membrane. (**D**) The ultrastructural view of Civatte body. The low magnification image is at 2,500× magnification. Scale bar: 5 μm. The localized magnified images are at 1,0000× magnification. Scale bar: 1 μm. A, apoptotic keratinocyte; KC, keratinocyte; C, Civatte body; Orange star, keratin filament; Blue arrow, mitochondria. (**E**) Immunohistochemical images of CD8^+^ T cell staining (original magnification, ×400; scale bar: 25 μm). (**F**) Quantification of CD8^+^ T cells per high-power field (HPF). (**G**) Immunofluorescence staining for keratin 5 (KRT5, red) and TUNEL (green) (original magnification, ×200; scale bar: 50 μm). (**H**) Quantification of KRT5^+^TUNEL^+^ cells in lower epithelium per HPF. (**I**) Immunostaining of vehicle or OXA-treated mice for CD31 (red) and DAPI (blue; original magnification, ×200; scale bar: 50 μm). (**J**) Quantification of the vascular area in the vehicle or OXA-treated mice (*n* = 5/group); the area in the vehicle groups was set as “1”. White dashed lines indicate the epithelium-lamina propria junction in panels (**E** and **G**). N= 5 each group. Results are representative of 3 independent experiments. Statistical significance: **P < 0.01. A 2-tailed Student’s *t* test was used.

To model OLP in mice, mice were first sensitized with 3.0% OXA applied to the skin, followed by a topical challenge to the labial mucosa with either OXA or vehicle (ethanol) (Supplemental Figure 1A; supplemental material available online with this article; https://doi.org/10.1172/jci.insight.185380DS1). Five days after challenge, dermoscopic examination of the OXA-treated group revealed labial erythema, swelling, submucosal vascular dilation, and significantly increased lip thickness compared with controls (Figure 1A and Supplemental Figure 1, B and C). Histological analysis confirmed dense lymphocytic infiltration at the epithelium-lamina propria interface in OXA-treated mice, along with basal cell vacuolization (Figure 1B). Civatte bodies were observed in H&E-stained sections (Figure 1C). Transmission electron microscopy further identified Civatte bodies in the basal layer containing keratin filaments and swollen mitochondria, consistent with apoptotic keratinocyte remnants (Figure 1D). Additional ultrastructural changes included intracellular edema of basal keratinocytes, mitochondrial swelling, and basement membrane alterations (Supplemental Figure 2). IHC revealed accumulation of CD3^+^, CD4^+^, and CD8^+^ T cells at the epithelium–lamina propria junction in OXA-treated mice, a pattern absent in vehicle controls. Representative high-power images of CD8^+^ T cell infiltration are shown in Figure 1E, with both low- and high-power images of CD3^+^ and CD4^+^ T cells presented in Supplemental Figure 3. Quantification confirmed a significant increase in CD8^+^ T cells per high-power field in OLP-like lesions (Figure 1F). Increased apoptotic activity was evidenced by TUNEL and cleaved-caspase 3 staining (Figure 1, G and H, and Supplemental Figure 4), which demonstrated a higher number of apoptotic cells at the epithelium-lamina propria interface in OXA-treated mice. In addition, vessel dilation within OLP-like lesions was confirmed by CD31 immunofluorescence, reflecting local vascular remodeling and inflammatory activation, with quantification showing a significantly larger vascular area in OXA-treated mice compared with controls (Figure 1, I and J). Collectively, these findings establish an OXA-induced mouse model that replicates key histopathological features of human OLP.

### Transcriptome analysis reveals similarities between OXA-induced OLP lesions and human OLP.

**Figure 2:**
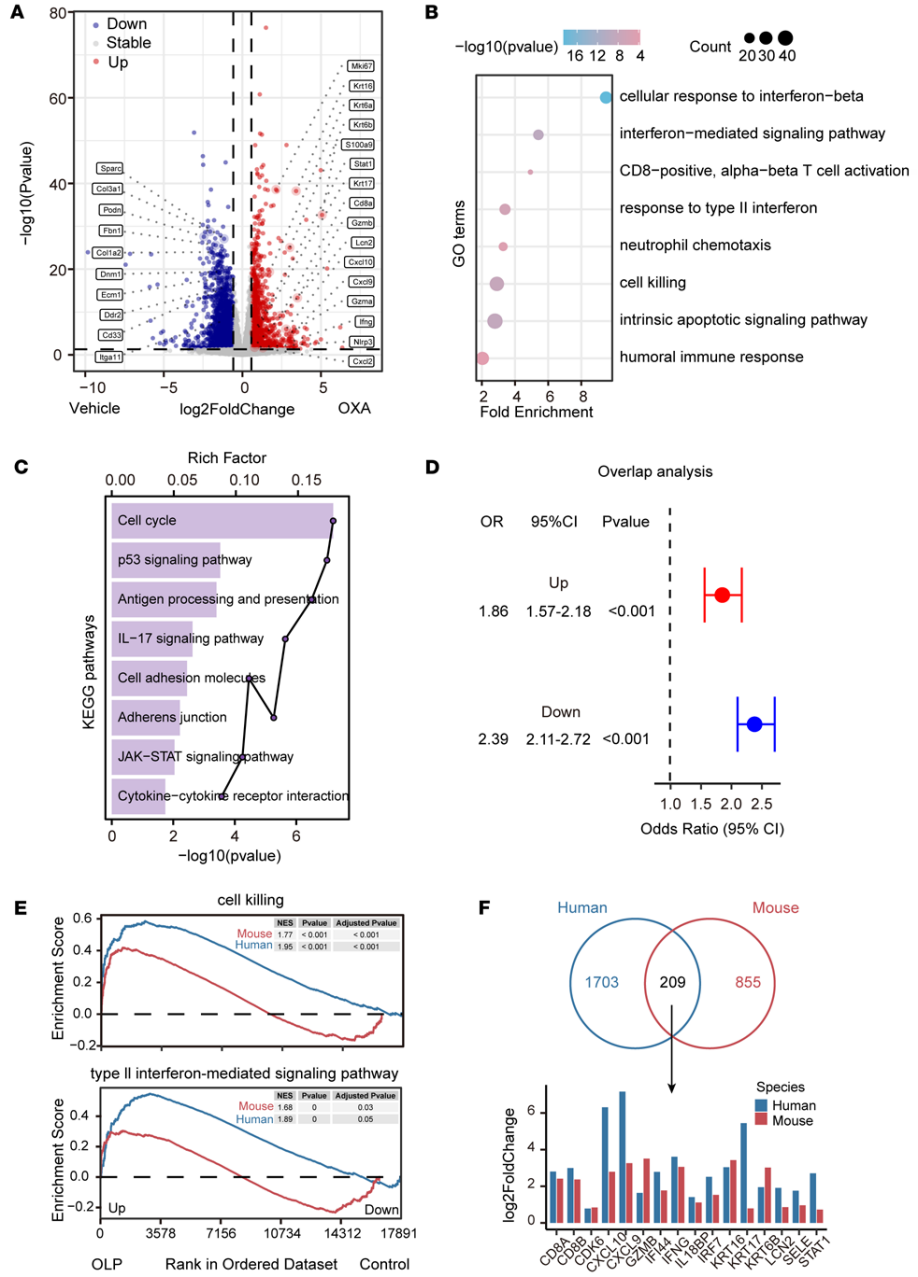
Comparative transcriptome analysis reveals similarities between mouse OLP-like lesions and human OLP lesions. (**A**) Volcano plots comparing gene expression in ethanol-treated control mice and OXA-induced OLP-like lesions (*n* = 6 for vehicle control group, *n* = 5 for OXA group per group). Representative significantly upregulated and downregulated genes are labeled. Dashed lines indicate thresholds for log_2_ fold change and adjusted *P* value. Genes not meeting these criteria are marked as “none.” (**B**) GO enrichment analysis of upregulated genes, highlighting IFN responses and immune-related pathways. Bubble size reflects the number of genes associated with each GO category, while bubble color represents the –log_10_(*P* value). (**C**) KEGG pathway enrichment analysis of upregulated genes, showing involvement in JAK/STAT signaling and immune pathways. The rich factor is represented by horizontal bars, while the –log_10_(*P* value) is depicted as connected points. (**D**) Overlap analysis of differentially expressed genes between mouse and human OLP datasets. Fisher’s exact test indicates significant concordance in both upregulated and downregulated genes; odds ratios (OR), 95% CI, and *P* values are shown. (**E**) Gene Set Enrichment Analysis (GSEA) demonstrating enrichment of “cell killing” and “type II interferon-mediated signaling pathway” in both human OLP lesions and OLP-like mouse lesions (left), compared with the control group (right). (**F**) Venn diagram showing overlapping upregulated genes between human and mouse datasets, with corresponding expression levels of representative genes displayed in a bar plot.

To explore the transcriptomic changes in OLP-like lesions, we performed bulk RNA-Seq of OXA-treated labial mucosa and vehicle-applied labial mucosa (*n* = 4). Differential expression analysis revealed significant upregulation of genes such as *Ifng*, *Cxcl9*, *Cxcl10*, *Cd8a*, *Gzmb*, *S100a9*, *Mki67*, *Nlrp3*, and *Stat1* in OXA-induced OLP-like lesions compared with controls, as shown in volcano plots (Figure 2A). To corroborate these findings, immunofluorescence staining demonstrated a significant increase in Ki67^+^ basal layer keratinocytes in OLP-like lesions, supporting enhanced epithelial cell proliferation (Supplemental Figure 6). qPCR further confirmed elevated expression of proinflammatory cytokines and chemokines, including *Ifng*, *Il1b*, *Il6*, *Cxcl9*, *Cxcl10*, *Cxcl16*, *S100a8*, and *S100a9* in OXA-treated mice compared with controls (Supplemental Figure 7). To characterize the biological functions of these upregulated genes, Gene Ontology (GO) and Kyoto Encyclopedia of Genes and Genomes (KEGG) pathway enrichment analyses were conducted. The upregulated genes were predominantly enriched in interferon-mediated signaling pathways, CD8^+^, α-β T cell activation, cell killing, intrinsic apoptotic signaling pathway, IL-17 signaling pathway, and JAK/STAT signaling pathways, consistent with immune activation in OXA-induced lesions (Figure 2, B and C).

To assess the translational relevance of the OXA-induced model, we compared its transcriptomic profile with human OLP lesions. Fisher’s exact test demonstrated significant concordance between mouse and human datasets, both for upregulated genes (OR = 1.86, 95% CI = 1.57–2.18, *P* < 0.001) and downregulated genes (OR = 2.39, 95% CI = 2.11–2.72, *P* < 0.001) (Figure 2D). Gene set enrichment analysis (GSEA) further revealed shared enrichment of critical immune pathways, including “cell killing” and “type II interferon-mediated signaling pathway,” in both datasets (Figure 2E). Notably, central inflammatory genes such as GZMB, CD8A, CD8B, IFNG, CXCL10, and CXCL9 were consistently upregulated across species (Figure 2F). Together, these data demonstrate a high degree of transcriptomic similarity between OXA-induced OLP-like lesions and human OLP, supporting the validity of this model in capturing shared immunopathogenic mechanisms.

### CD8^+^ T cells are essential for the development of OLP-like lesions.

**Figure 3:**
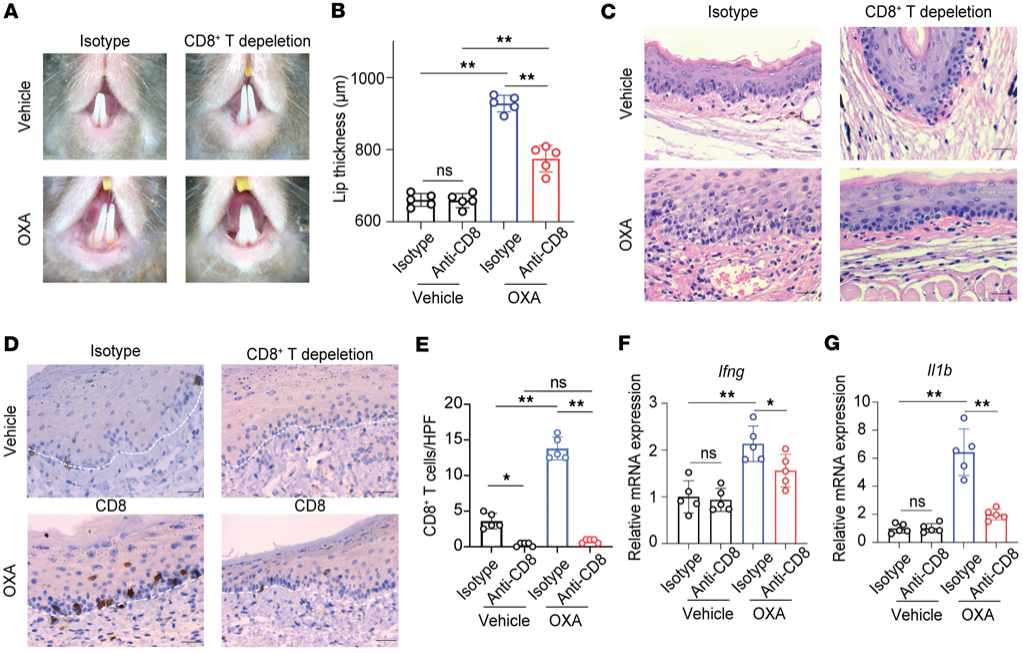
CD8^+^ T cells are essential for the development of an OLP-like model. CD8^+^ T cells were depleted in vivo through i.p. injections of neutralizing antibodies on days –1, 2, and 4 during the elicitation phase. (**A**) Dermoscopic images of lesions on Day 5 in ethanol-treated controls, OXA-treated isotype control mice, and CD8^+^ T cell–depleted mice. (**B**) Lip thickness measurements on Day 5 across the groups. (**C**) Representative H&E-stained sections of lesions from all groups (original magnification, ×400; scale bar: 25 μm). (**D**) IHC staining for CD8^+^ T cells (original magnification, ×400; scale bar: 25 μm). (**E**) Quantification of CD8^+^ T cells per high-power field (HPF). (**F** and **G**) Relative mRNA expression levels of *Ifng* and *Il1b* as determined by qPCR. White dashed lines indicate the epithelium-lamina propria junction in **D**. Data are presented as mean ± SD (*n* = 5/group). Results are representative of 3 independent experiments. *P < 0.05; **P < 0.01. A 1-way ANOVA with a Tukey post hoc test was used.

To investigate the role of CD8^+^ T cells in the development of OLP-like lesions, we depleted these cells in vivo using neutralizing antibodies administered i.p. on days –1, 2, and 4 of the elicitation phase. CD8^+^ T cell depletion significantly reduced their frequency in peripheral blood and cervical draining lymph nodes (Supplemental Figure 8). On day 5, dermoscopic examination revealed reduced erythema in the CD8^+^ T cell–depleted group compared with the isotype control group (Figure 3A). Measurements of lip thickness showed a significant reduction in the depletion group, further supporting the attenuated lesion severity (Figure 3B). Histological analysis with H&E staining showed diminished lymphocytic infiltration at the epithelium-lamina propria junction in CD8^+^ T cell–depleted mice (Figure 3C). IHC staining confirmed a significantly decrease in CD8^+^ T cells at the lesion site in the depletion group (Figure 3, D and E). qPCR analysis of the lesion tissue revealed lower mRNA expression levels of proinflammatory cytokines *Ifng*, *Il1b*, *cxcl9*, and *cxcl10* in CD8^+^ T cell–depleted mice compared with controls (Figure 3, F and G, and Supplemental Figure 9A). Consistently, KRT5 and TUNEL costaining indicated fewer apoptotic keratinocytes in the CD8^+^ T cell–depleted group, correlating with reduced tissue damage (Supplemental Figure 9B). These results collectively highlight the indispensable role of CD8^+^ T cells in driving the development of OLP-like lesions in this mouse model.

### IFN-γ is crucial for the development and severity of OLP-like lesions.

**Figure 4:**
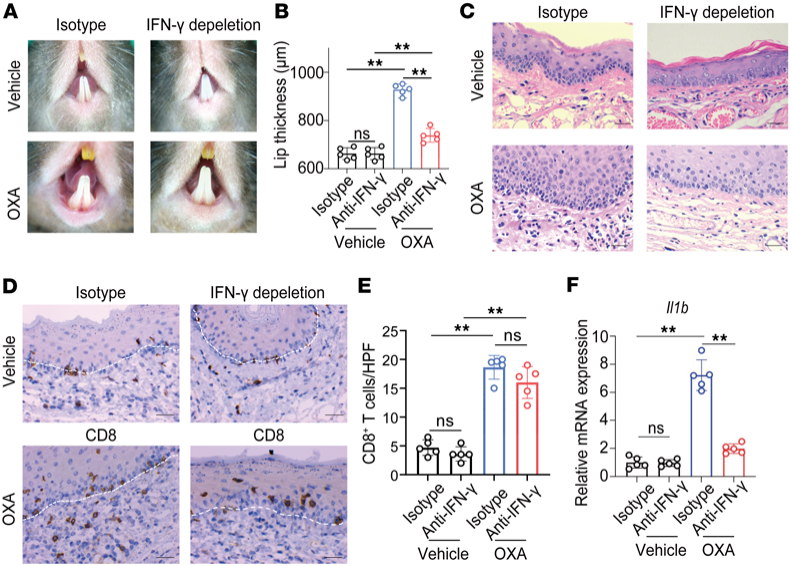
Neutralization of IFN-γ ameliorates OLP-like lesions. IFN-γ was neutralized in vivo by administering neutralizing antibodies every third day during the elicitation phase. (**A**) Dermoscopic images of lesions on day 5 in the isotype control group and the IFN-γ–neutralized group. (**B**) Lip thickness measurements on day 5 in both groups. (**C**) Representative H&E-stained sections of lesions (original magnification, ×400; scale bar: 25 μm). (**D**) Immunohistochemical staining for CD8^+^ T cells (original magnification, ×400; scale bar: 25 μm). (**E**) Quantification of CD8^+^ T cells per high-power field (HPF). (**F**) Relative mRNA expression of *Il1b* as determined by qPCR. White dashed lines indicate the epithelium-lamina propria junction in **D**. Data are presented as mean ± SD (*n* = 5/group). Results are representative of 2 independent experiments. Statistical significance: ns, not significant; **P < 0.01. A 1-way ANOVA with a Tukey post hoc test was used.

To evaluate the role of IFN-γ in the development of OLP-like lesions, we administered neutralizing antibodies targeting IFN-γ every third day during the elicitation phase. By day 5, dermoscopic imaging revealed significant improvements in lesion appearance in the IFN-γ–depleted group compared with the isotype control group (Figure 4A). Lip thickness measurements confirmed a significantly reduction in the IFN-γ–depleted group (Figure 4B). Histological analysis demonstrated decreased lymphocytic infiltration in these mice (Figure 4C). IHC staining for CD8^+^ T cells showed no significant difference in the number of these cells between the 2 groups (Figure 4, D and E). qPCR analysis indicated reduced mRNA expression of the proinflammatory cytokine *Il1b* in IFN-γ–depleted mice compared with controls (Figure 4F). The expression levels of *Cxcl9* and *Cxcl10* were not significantly affected by IFN-γ depletion (Supplemental Figure 10A). KRT5 and TUNEL costaining further revealed a significant reduction in the number of apoptotic keratinocytes in the IFN-γ–depleted group (Supplemental Figure 10B). Together with previous in vitro findings (8), these results demonstrate that IFN-γ plays a pivotal role in the development of OLP-like lesions, and its neutralization effectively mitigates disease pathology.

**Figure 5:**
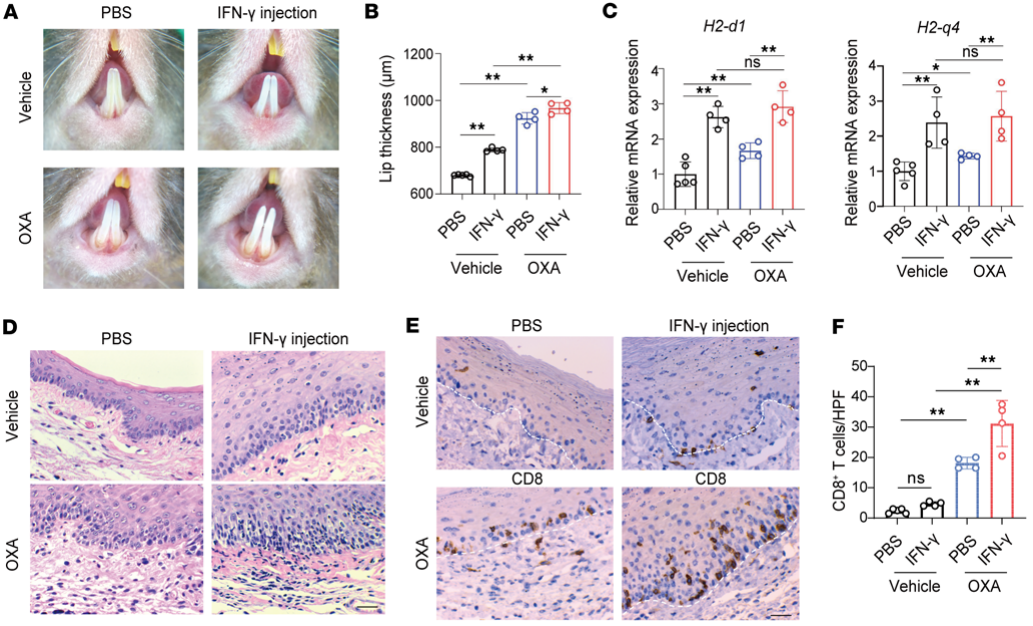
Local IFN-γ priming exacerbates OLP-like lesions by upregulating MHC I expression. (**A**) Dermoscopic images of lesions on day 5 in the OXA and vehicle groups, with or without IFN-γ priming. (**B**) Lip thickness measurements on day 5 across the groups (*n* = 4–5/group). (**C**) qPCR analysis of relative expression levels of *H2-d1* and *H2-q4* (*n* = 4–5/group). (**D**) Representative H&E-stained sections of lesions (original magnification, ×400; scale bar: 25 μm). (**E**) Representative images of IHC staining for CD8^+^ T cells (original magnification, ×400; scale bar: 25 μm). (**F**) Quantification of CD8^+^ T cells per high-power field (HPF, *n* = 4–5 each group). White dashed lines indicate the epithelium-lamina propria junction in **E**. Data are presented as mean ± SD. Results are representative of 2 independent experiments. *P < 0.05; **P < 0.01. A 1-way ANOVA with a Tukey post hoc test was used.

To investigate the effect of IFN-γ priming on disease severity, recombinant IFN-γ was locally injected into the inner labial mucosa on days –1, 2, and 4 of the elicitation phase. By day 5, dermoscopic images showed exacerbated lesion severity in OXA-treated mice primed with IFN-γ compared with PBS-treated controls (Figure 5A). Lip thickness measurements further confirmed significant increases in lesion severity in the IFN-γ–primed group (Figure 5B). qPCR analysis revealed upregulation of MHC I–related genes *H2-d1* and *H2-q4* in IFN-γ–primed OXA-treated mice compared with those without priming; however, these levels did not exceed those observed in IFN-γ–primed, vehicle-treated mice (Figure 5C). Histological analysis showed increased lymphocytic infiltration and basal cell vacuolization in the OXA-treated, IFN-γ–primed group (Figure 5D). qPCR analysis showed a significant upregulation of *Cxcl9* and *Cxcl10* in the IFN-γ–primed group (Supplemental Figure 11A). IHC staining further demonstrated significantly higher CD8^+^ T cell infiltration in IFN-γ–primed mice (Figure 5, E and F). Additionally, KRT5 and TUNEL costaining revealed an increased number of apoptotic keratinocytes in IFN-γ–primed mice (Supplemental Figure 11B). These findings suggest that IFN-γ priming exacerbates OLP-like lesions by upregulating MHC I expression, which intensifies CD8^+^ T cell–mediated pathology.

### Absence of Langerhans cells exacerbates OLP-like lesions.

**Figure 6:**
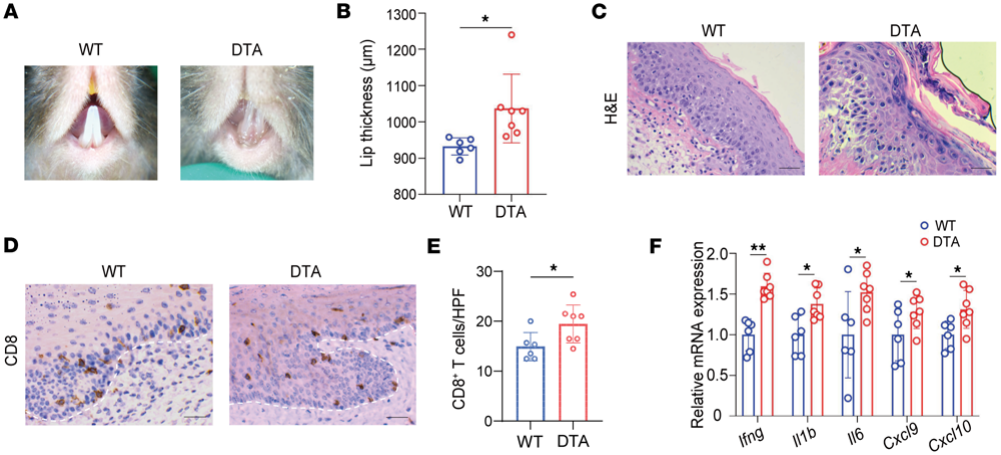
Absence of Langerhans cells exacerbates OLP-like lesions. (**A**) Dermoscopic images of lesions on day 5 in WT and hLangerin-DTA mice. (**B**) Lip thickness measurements on day 5 in both groups. (**C**) Representative H&E-stained sections of lesions (original magnification, ×400; scale bar: 25 μm). (**D**) IHC staining for CD8^+^ T cells, with representative images (original magnification, ×400; scale bar: 25 μm). (**E**) Quantification of CD8^+^ T cells per high-power field (HPF). (**F**) Relative mRNA expression of *Ifng*, *Il1b*, *Il6*, *Cxcl9*, and *Cxcl10* as determined by qPCR. White dashed lines indicate the epithelium-lamina propria junction in **D**. Data are presented as mean ± SD (*n* = 6–7/group). Results are representative of 2 independent experiments. *P < 0.05; **P < 0.01. A 2-tailed Student’s *t* test was used.

Langerhans cells (LCs) are known to function as antigen-presenting cells and negative regulators of local immune responses (24), but their role in OLP pathogenesis remains unclear. To investigate their involvement in OLP-like lesions, we compared WT mice to hLangerin-DTA mice, which lack LCs. By day 5, dermoscopic imaging showed more severe lesions in hLangerin-DTA mice compared with WT controls (Figure 6A). Lip thickness measurements confirmed significantly greater inflammation in the LC-deficient group (Figure 6B). Histological analysis with H&E staining revealed increased interface inflammation in hLangerin-DTA mice (Figure 6C). IHC staining demonstrated significantly higher CD8^+^ T cell infiltration in the absence of LCs (Figure 6, D and E). qPCR analysis indicated elevated mRNA expression levels of *Ifng*, *Il1b*, *Il6*, *Cxcl9*, and *Cxcl10* in hLangerin-DTA mice compared with WT controls (Figure 6F). Moreover, KRT5 and TUNEL costaining showed increased apoptotic cell counts in the LC-deficient group (Supplemental Figure 12). These findings suggest that the absence of LCs exacerbates OLP-like lesions, underscoring their potential protective role in modulating immune responses in this model.

### Systematic or topical JAK inhibitor ameliorates the OLP-like lesions.

**Figure 7:**
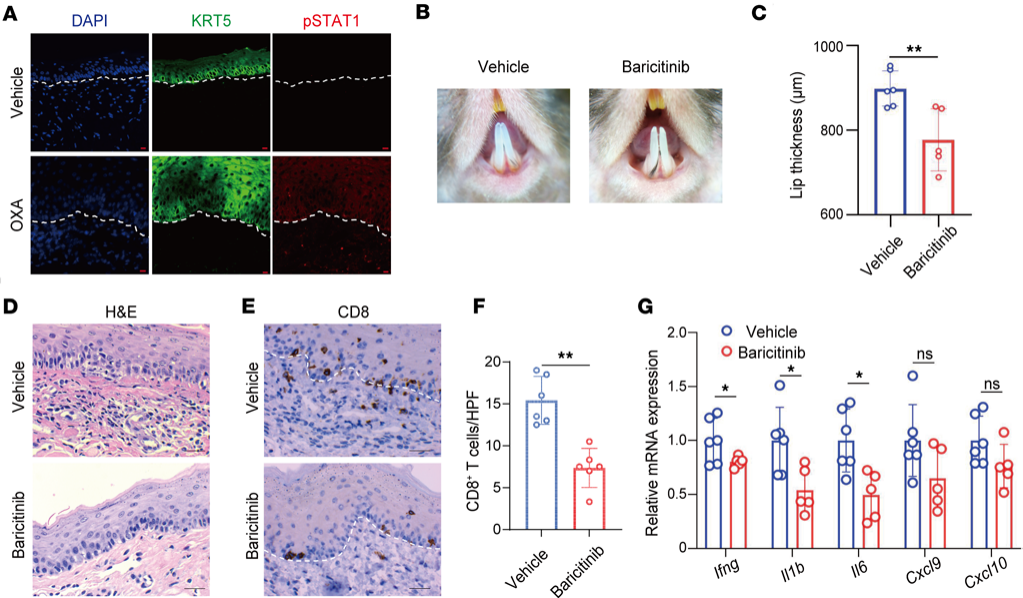
The JAK inhibitor baricitinib ameliorates OLP-like lesions. (**A**) Representative immunofluorescence images showing costaining of phosphorylated STAT1 (pSTAT1) and keratin 5 (KRT5) in vehicle-treated and OXA-treated mice (original magnification, ×400; scale bar: 10 μm). (**B**) Dermoscopic images of lesions on day 5 in the corn oil control group and the baricitinib-treated group. (**C**) Lip thickness measurements on day 5 across the groups. (**D**) Representative H&E-stained sections of lesions (original magnification, ×400; scale bar: 25 μm). (**E**) IHC staining for CD8^+^ T cells, with representative images (original magnification, ×400; scale bar: 25 μm). (**F**) Quantification of CD8^+^ T cells per high-power field (HPF). (**G**) Relative mRNA expression levels of *Ifng*, *Il1b*, *Il6*, *Cxcl9*, and *Cxcl10* as determined by qPCR. White dashed lines indicate the epithelium-lamina propria junction in **A** and **E**. Data are presented as mean ± SD (n = 5–6/group). Results are representative of 2 independent experiments. *P < 0.05; **P < 0.01. A 2-tailed Student’s *t* test was used.

The JAK pathway has been implicated in the pathogenesis of lichen planus (10). To explore its role in the OLP-like mouse model, we performed immunofluorescence staining for phosphorylated STAT1 (pSTAT1) on tissue sections from normal mucosa and OLP-like lesions, which showed significantly elevated pSTAT1 expression in keratinocytes within OLP-like lesions (Figure 7A), suggesting JAK pathway activation in the disease process. To evaluate the therapeutic potential of JAK inhibition, mice were treated with baricitinib phosphate (5 mg/kg) via daily oral gavage during the elicitation phase. By day 5, dermoscopic imaging revealed improvement in lesion severity in baricitinib-treated mice compared with controls (Figure 7B). Lip thickness measurements confirmed a significant reduction in the treated group (Figure 7C). Histological analysis demonstrated reduced lymphocytic infiltration (Figure 7D), while IHC staining showed a significant decrease in CD8^+^ T cell infiltration in baricitinib-treated lesions (Figure 7, E and F). qPCR analysis further revealed lower mRNA expression levels of proinflammatory cytokines *Ifng*, *Il1b*, and *Il6* in baricitinib-treated mice compared with controls (Figure 7G). Topical application of the JAK inhibitor ruxolitinib also alleviated the OLP-like inflammatory phenotype (Supplemental Figure 13, A and B). However, unlike baricitinib, ruxolitinib did not significantly reduce CD8^+^ T cell infiltration in the lesions, as shown by IHC staining (Supplemental Figure 13, C and D). qPCR analysis indicated reduced mRNA expression of *Ifng* and *Il1b* in the ruxolitinib-treated group (Supplemental Figure 14, E and F). These findings demonstrate that both systemic or topical administration of JAK inhibitors could effectively ameliorate OLP-like lesions.

## Discussion

Animal models are indispensable tools in biomedical research, providing critical insights into disease mechanisms and enabling the development of novel therapies. Building on previous efforts to replicate human OLP in animal models (13), this study introduces an approach employing OXA-induced delayed hypersensitivity to establish a mouse model of OLP, which successfully mirrors the core pathogenesis of OLP and reproduces key histopathological features observed in human lesions. Its simplicity and reproducibility make it a valuable tool for investigating disease mechanisms and evaluating potential therapeutic interventions.

Current understanding suggests that OLP is driven by reactive CD8^+^ T cells targeting antigens at the epithelial-lamina propria junction (4, 18). Our model employs OXA-induced type IV hypersensitivity to generate CD8^+^ T cells reactive to an OXA-epithelial protein complex. Type IV hypersensitivity consists of sensitization and elicitation phases (25, 26). During sensitization, OXA, a hapten, is applied to the abdominal skin, where it binds to local proteins, induces protein denaturation, and stimulates the production of antigen-specific CD8^+^ T cells. In the elicitation phase, applying OXA to the labial mucosa denatures epithelial proteins, triggering an autoreactive inflammatory response. This mechanism mirrors the pathogenesis of OLP. Previous research by Dunsche et al. on a mercury-induced OLP-like reaction in rats revealed some similar features but predominantly reflected local irritation (16). By contrast, mice provide genetic engineering versatility, enabling deeper exploration of OLP pathogenesis through transgenic models (27).

Pathologically, OLP is characterized by band-like infiltration of inflammatory cells in the lamina propria and liquefactive necrosis of basal keratinocytes (28). Our mouse model exhibits these hallmark features. Histological analysis reveals band-like inflammatory infiltration at the epithelial-lamina propria interface, with IHC and immunofluorescence confirming CD8^+^ T cell involvement. Additionally, basal keratinocyte death, a key feature of lichen planus, includes apoptosis and necrosis. TUNEL staining, cleaved caspase-3 staining, and transmission electron microscopy confirm keratinocyte apoptosis and necrosis near the basal membrane, consistent with human OLP and tissue-engineered models (29, 30). The small size of the mouse oral cavity poses challenges for gross observation. To address this, we used polarized light dermoscopy to eliminate saliva reflections, allowing visualization of erythema and edema, core clinical features of OLP (31). Thus, our OXA-induced model replicates key clinical and pathological features of OLP.

Previous studies have established the critical roles of CD8^+^ T cells and IFN-γ in the pathogenesis of OLP (32). In our study, we observed significantly elevated levels of chemokines *Cxcl9*, *Cxcl10*, and *Cxcl16* — key attractants of CD8^+^ T cells — in OLP-like lesions. These findings align with reports from human lichen planus studies (33–35). Furthermore, depleting CD8^+^ T cells in our mouse model resulted in a notable reduction in liquefied basal keratinocytes and TUNEL^+^ keratinocytes, reinforcing the role of CD8^+^ T cells in inducing keratinocyte apoptosis (5, 36). Building on our earlier research, which demonstrated that IFN-γ upregulates MHC I expression on keratinocytes and facilitates CD8^+^ T cell–mediated cytotoxicity (8), this study further confirms these findings in vivo. Depletion of CD8^+^ T cells or IFN-γ significantly ameliorated OLP-like lesions, while local injection of IFN-γ exacerbated disease manifestations by upregulating MHC I molecules. These observations are consistent with previous in vitro studies showing that T cell clones from patients with lichen planus exhibit MHC I–dependent cytotoxic activity against autologous keratinocytes (37). We also demonstrated that IFN-γ plays a critical role in CD8^+^ T cell recruitment to the basal epidermal layer. IFN-γ depletion reduced CD8^+^ T cell infiltration, while IFN-γ priming significantly increased their numbers. These findings align with our previous research (23). Interestingly, the role of IFN-γ may vary depending on disease context. Phuc et al. (17) recently reported a zinc deficiency– and Escherichia coli–driven OLP-like model in which IFN-γ blockade enhanced the local Th17 response and exacerbated erosive lesions (17). The distinct outcomes likely reflect differences in induction mechanisms, as their model is infection driven, whereas ours is primarily mediated by CD8^+^ T cells and IFN-γ. Given that microbial dysbiosis is often observed in patients with OLP and may shape disease pathology (38), diverse OLP-like models are valuable for dissecting different pathogenic pathways and therapeutic strategies.

Our OLP-like mouse model also provides a platform for therapeutic exploration. Using this model, we recently demonstrated that apoptotic vesicles derived from stem cells of human exfoliated deciduous teeth (SHED) significantly alleviated OLP-like lesions (39). Here, we evaluated the therapeutic potential of JAK inhibitors, which have shown promise in recent OLP studies (10, 40–42). Using our mouse model, we found that baricitinib and ruxolitinib effectively mitigated OLP-like manifestations. These findings may extend beyond OLP, as other diseases involving cytotoxic CD8^+^ T cell–mediated lichenoid reactions, such as chronic lung allograft dysfunction (43), also involve JAK/STAT pathway activation. Thus, JAK inhibitors may hold therapeutic potential for graft-versus-host disease and related conditions (44).

In addition to T cells, this study examined the role of LCs in OLP. LCs residing in the oral mucosa capture antigens and present them to T cells (4, 24, 45, 46). While previous research indicates no significant increase in epithelial LCs in OLP lesions (47), we observed a rise in langerin^+^ cells within the lamina propria. The role of LCs in the pathogenesis of OLP is difficult to investigate through in vitro experiments (29). Using hLangerin-DTA mice, which lack epithelial LCs but retain langerin^+^ dendritic cells under inflammatory conditions (48), we demonstrated that epithelial LCs play an immunosuppressive role in OLP. This effect may be linked to neuroimmune crosstalk; recent evidence suggests that LCs maintain epidermal MrgprD^+^ sensory neurons, which tonically suppress mast cell activation (49). Given the parallels between OXA-induced OLP and contact hypersensitivity, LC deficiency in oral mucosa may similarly disrupt neuron–mast cell interactions. This finding aligns with findings in *P*. *gingivalis*–induced alveolar bone loss (50), oral squamous cell carcinoma (51), photosensitivity (52), and psoriasis (53).

Despite its advantages, our model has limitations. Although it recapitulates key histopathological and immunological features of OLP, it does not fully reflect the complexity of human disease. Notably, malignant transformation to oral squamous cell carcinoma and clinical features such as Wickham striae were not observed (3, 17, 54, 55). These differences may be attributable to anatomical variations, as the predominantly keratinized oral mucosa in mice contrasts with the nonkeratinized mucosa commonly affected in humans. Moreover, while the model effectively captures acute T cell–mediated inflammation, it does not encompass the chronic relapsing course, microbial influences, or genetic predispositions characteristic of human OLP (56, 57). In this regard, short-term OXA application (5 elicitations) in our study generates a type 1–dominated immune response, consistent with our RNA-Seq data, the absence of eosinophilia, and the effects of IFN-γ neutralization. By contrast, repeated OXA exposure (≥10 elicitations) is widely employed as a model of type 2–biased conditions such as atopic dermatitis; our regimen therefore differs immunologically and histopathologically from such chronic protocols, which typically induce type 2 features. These limitations should be considered when interpreting findings, and future studies are warranted to refine the model.

In summary, the OLP-like mouse model established here aligns closely with the core pathogenic mechanisms and pathological features of OLP. Lichen planus is a prototypical interface dermatitis and a representative Th1-driven dermatosis. These categories encompass several related conditions, including chronic cutaneous graft-versus-host disease, cutaneous lupus, and cutaneous immune-related adverse events. Developing animal models for lichen planus not only advances our understanding of OLP but also provides valuable insights into these related diseases.

## Methods

### Sex as a biological variable.

Our study examined male and female animals, and similar findings were observed for both sexes.

### Animals.

In this study, C57BL/6 mice were obtained from the Fourth Military University for experimental use. Human langerin (CD207) diphtheria toxin gene (DTA) mice from The Jackson Laboratory (stock no. 017949) were housed in specific pathogen-free conditions as we previously reported (53, 58). All mice were 10- to 14-week-old female or male mice and maintained on a 12-hour light/dark cycle.

### OLP-like model and pharmacological treatment.

Mice were sensitized by epicutaneous application of 100 μL of 3.0% OXA (dissolved in anhydrous ethanol, Sigma-Aldrich, St. Louis, MO) on shaved abdomen. Five days later, labial oral mucosal was challenged with 15 μL of 1% OXA or ethanol for 5 consecutive days. Before harvesting the lip, the dermatoscopic appearances of the lesions were recorded using a dermatoscope (ILLUCO IDS 1100; South Korea) as we previously described (59). After harvesting the lip, the lip thickness was measured using an engineer’s micrometer caliper. Labial mucosa were harvested under stereomicroscopy (Supplemental Figure 1D). To deplete CD8^+^ T cells, 200 μg of anti-CD8 (clone 2.43, BioXCell) was injected i.p. on day –1, 2, and 4 in the elicitation phase. For in vivo blocking of IFN-γ, mice were injected i.p. every third day with 200 μg of InVivoMAb anti–mouse IFN-γ (clone XMG1.2, catalog BE0055 BioXCell). Injection of the same amount of IgG antibodies (rat IgG2 isotype control for anti-mouse CD8, clone LTF-2, catalog BE0090, BioXCell; rat IgG1 isotype control for anti-mouse IFN-γ, clone HRPN, catalog BE0088, BioXCell) was used in the control group. To explore the IFN-γ priming effects, on day –1, 2, and 4 in the elicitation phase, 20 μL recombinant mouse IFN-γ (20 ng/mL, SinoBio, China) was locally injected into the labial mucosal using a 32G syringe. For systematic JAK inhibition, mice were intragastric administration with 5 mg/kg Baricitinib phosphate (dissolved in 10% DMSO and 90% corn oil, MedChemExpress, China) once daily from the day before elicitation to the fifth day. For local JAK inhibition, mice were topically applied with 1.5% ruxolitinib (dissolved in DMSO, TargetMol, China) once daily 20 minutes after OXA painting during the elicitation phase.

### Electron microscopy.

Ultrathin about 70 nUltra-thin (about 70 nm) electron microscopy samples from the mice were prepared and osmium stained as we described previously (60). The images were collected using transmission electron microscopy at 120kV (FEI Technai G2; Thermo Fisher Scientific).

### Flow cytometry.

To assess the efficacy of the CD8 depletion antibody, we collected peripheral blood and draining lymph nodes from mice one day after the injection of the CD8 neutralizing antibody. Single-cell suspensions were prepared and compared with an isotype control group. The following fluorophore-labeled monoclonal antibodies were used. CD3e (clone 145-2C11, catalog100306, BioLegend, San Diego, CA, USA) and CD8 (clone 53-6.7, catalog 100762, BioLegend, San Diego, CA, USA) antibodies were used. Flow cytometry was performed using the FACS Calibur system (BD Biosciences, Franklin Lakes, NJ, USA), and data were analyzed with FlowJo software (FlowJo v10; TreeStar, Ashland, OR, USA).

### Histology, IHC, and immunofluorescence.

Briefly, lip tissues were collected and fixed in 10% formalin overnight. After fixation, tissues were embedded in paraffin blocks, sectioned at 4 μm thickness, and stained with H&E. For the number of Keratin 5^+^TUNEL^+^ cells, TUNEL^+^ keratinocytes in the lower epithelium were counted in each high-power field under the microscope, excluding those in the granular layer of the epidermis. Recombinant anti-CD3 antibody (1:500, GB11014, Servicebio), anti-CD4 antibody (1:400, GB15064, Servicebio), and anti-CD8α antibody (1:400, GB15068, Servicebio) were used in IHC staining. For the detection of apoptotic cells in tissue sections, TUNEL staining with keratin 5 costaining was performed according to manufacturer protocol (DeadEnd Fluorometric TUNEL system, Promega). Briefly, paraffin-embedded skin slides were dewaxed and rehydrated following standard protocols and then treated with proteinase K solution. Subsequently, the slides were incubated with the TUNEL reaction mixture in a humidified chamber, followed by washing with PBS. Finally, the slides were mounted with DAPI (G1012, Servicebio). For immunofluorescence, rabbit anti-keratin 5 antibody (1:1000, GB111246, Servicebio), rabbit anti-cleaved-caspase 3 antibody (1:500, GB11532, Servicebio), mouse anti-CD31 antibodies (1:100, ab9498, Abcam), rabbit anti-granzyme b antibody (1:3000, ab255598, Abcam), rabbit anti-perforin antibody (1:400, 31647, Cell Signaling Technology), and rabbit anti-phospho-STAT1 (Tyr701) antibody (1:400, 9167, Cell Signaling Technology) were used as primary antibodies. Goat anti-mouse IgG (Alexa Fluor 647, 1:500, ab150115, Abcam), Goat anti-rabbit IgG (Alexa Fluor 594, 1:500, ab150080, Abcam), and Goat anti-rabbit IgG (Alexa Fluor 488, 1:500, ab150077, Abcam) were used as secondary antibodies. For the analysis of relative vascular area, the total cross-sectional area of blood vessels within the lamina propria was quantified over a defined length of epithelium in each tissue section. The mean vascular area of control group mouse sections was set as 1, and the experimental values were normalized to this reference. For the quantification of CD8^+^ T cells or keratin5^+^TUNEL^+^ keratinocytes, we evaluated 3–5 high-power fields (HPFs) along the labial mucosa epithelium-lamina propria junction to ensure adequate sampling and accuracy. Staining was quantified by averaging the results from these 3–5 HPFs for each tissue section, providing a single representative value for each sample.

### Purification of RNA and RT-PCR.

Total RNA was purified using TRIzol Reagent (Invitrogen, Life Technologies, CA, USA) according to the manufacturer’s protocol. cDNA was synthesized from 2 μg of total RNA using Prime Script RT Master Mix (Takara, Dalian, China), and quantitative real-time PCR was performed on CFX384 Real-Time PCR detection system (Bio-Rad, CA, USA) using SYBR Premix Ex Taq II (Takara, Dalian, China). The cycling conditions were as follows: 95°C for 1 minute, followed by 40 cycles of 95°C for 15 seconds, 60°C for 15 seconds, and 72°C for 30 seconds, followed by dissociation curve analysis to verify the amplification of a single product. The primers were synthesized by Sangon Inc (Shanghai, China). All values were normalized to the levels of Hprt. Primer sets are shown in Supplemental Table 1.

### RNA-Seq and analysis.

RNA-Seq of labial mucosa samples from OXA-treated mice and vehicle-treated mice was conducted (Biomarker Technologies Co., Ltd, Beijing, China). RNA quality was assessed on an Agilent 2100 Bioanalyzer (Agilent Technologies, Palo Alto, CA, USA) and checked using RNase free agarose gel electrophoresis. After total RNA was extracted, eukaryotic mRNA was enriched by Oligo(dT) beads. Then the enriched mRNA was fragmented into short fragments using fragmentation buffer and reverse transcripted into cDNA with random primers. Second-strand cDNA was synthesized by DNA polymerase I, RNase H, dNTP, and buffer. Then the cDNA fragments were purified with QiaQuick PCR extraction kit (Qiagen, Venlo, Netherlands), end repaired, poly(A) added, and ligated to Illumina sequencing adapters. The ligation products were size selected by agarose gel electrophoresis, PCR amplified, and sequenced using Illumina Novaseq6000. The differential expression analyses of patient RNA-Seq datasets utilized in this study were performed using DEseq2 (version 1.44.0). Differentially expressed genes were defined by a log_2_ fold change value greater than 1 or less than −1 and adjusted *P* value lower than 0.05. The transcriptomic profiling datasets for OLP lesions and healthy controls have been described previously (https://bigd.big.ac.cn/; accession no. PRJCA017733). Differentially expressed genes were filtered for GO and KEGG enrichment analysis. Biological process GO enrichment analyses were performed separately on the set of differentially upregulated genes (a log_2_ fold change value greater than 1) common to OLP mouse lesions and lichen planus patient lesions using DAVID database. The resulting GO or KEGG terms with an adjusted *P* < 0.05 were considered significantly enriched. To assess transcriptional similarity between human OLP lesions and our mouse OLP-like lesions, we performed a fisher exact test for both significantly upregulated and downregulated genes from both species (adjusted *P* < 0.01 and log_2_ fold change > 0.25). Bonferroni-adjusted statistics are displayed in Figure 2D. Moreover, GSEA of OLP-related pathways including “cell killing” and “type II interferon-mediated signaling pathway” was done. The expression changes of core genes enriched among the upregulated genes in both species, which are implicated in driving OLP pathogenesis — including CD8A, CD8B, CXCL10, CXCL9, GZMB, IFNG, STAT1, and others — were analyzed. Custom codes are fully available upon request.

### Statistics.

Data are shown as mean ± SD. A 2-tailed Student’s *t* test or 1-way ANOVA with a Tukey post hoc test was employed to determine the statistical significance of the experimental data. The level of significance was set at *P* < 0.05.

### Study approval.

Animal experiments were approved by the IACUC of the university (Approval no. kq-2023-115) and were conducted in compliance with the guidelines outlined in the *Guide for the Care and Use of Laboratory Animals* (National Academies Press, 2011).

### Data availability.

The RNA-Seq data have been deposited at BIG Data Center (https://bigd.big.ac.cn/), Beijing Institute of Genomics, Chinese Academy of Sciences, with an accession no. of PRJCA044192. Values for all data points in graphs are reported in the Supporting Data Values file.

## Author contributions

Conceptualization was contributed by ZZ, TY, PP, KL, and SS. Investigation and laboratory work were contributed by ZZ, TY, PP, KL, WQ, CZ, SW, YW, MW, and HG. Data curation and statistics were contributed by PP, KL, CZ, ED, MF, HG, WY, SS, and QL. Original draft preparation and writing, review, and editing of the manuscript were contributed by ZZ, TY, WQ, SW, YW, MW, ED, SS, and QL. Project administration was contributed by YW, SS, and QL. Funding acquisition was contributed by ZZ and QL. Co–first authors ZZ, TY, and PP contributed equally to all experiments in this study. ZZ is listed first in the authorship list because of ZZ’s greater contribution to acquiring grant funding.

## Supplementary Material

Supplemental data

Supporting data values

## Version 1 12/11/2025

In-Press Preview

## Version 2 02/09/2026

Electronic publication
