# Supplementary material for: An oral lichen planus–like mouse model driven by IFN-γ signaling and cytotoxic CD8+ T cells
Source: JCI Insight. 2025 Dec 11;11(3):e185380. doi: 10.1172/jci.insight.185380 (PMC12892915; doi:10.1172/jci.insight.185380)
Supplement: Supplemental data [file jciinsight-11-185380-s008.pdf]

JCI insight

Supplementary files for

An Oral Lichen Planus-like Mouse Model Driven by IFN-γ signaling and cytotoxic CD8+ T Cells

Zhenlai Zhu *et al.*

*Corresponding author. Email: 857746654@qq.com (S.S.); liuqing@fmmu.edu.cn (Q.L.).

This PDF file includes:

Figs. S1 to S13

Table S1

Supplementary Figures and Figure legends

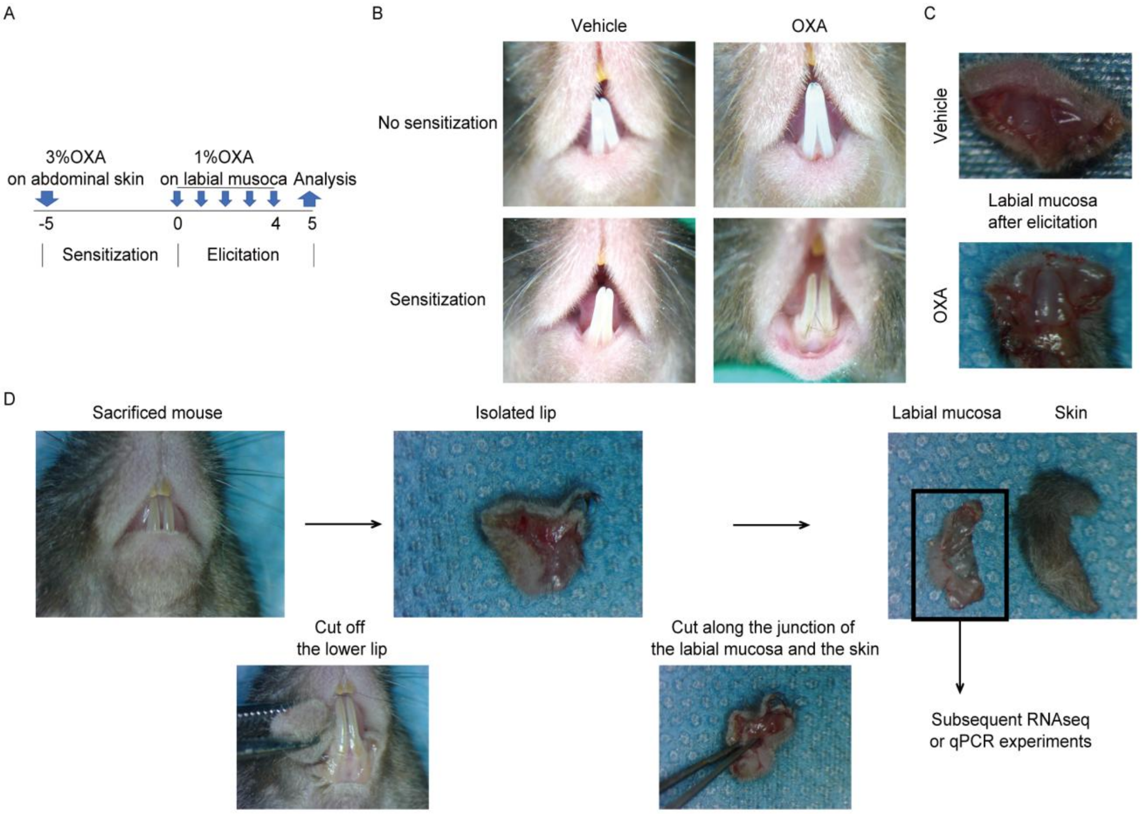

Supplemental Figure 1. Schematic diagram of the modeling process and the gross appearance of the labial mucosa before and after elicitation.

(A) Schematic representation of the modeling protocol. Mice were sensitized by epicutaneous application of 100 μl of 3.0% oxazolone (OXA) on shaved abdomen. Five days later, the labial oral mucosa was challenged with 15 μl of 1% OXA or vehicle (ethanol) for five consecutive days. (B) Dermoscopic appearances of the lip lesions on the fifth day after daily application of vehicle or OXA during the elicitation phase. (C) Stereomicroscopic views of the labial mucosa on the fifth day after daily application of vehicle or OXA. (D) Procedure for harvesting the labial mucosa. The results are representative of three independent experiments (for B and C).

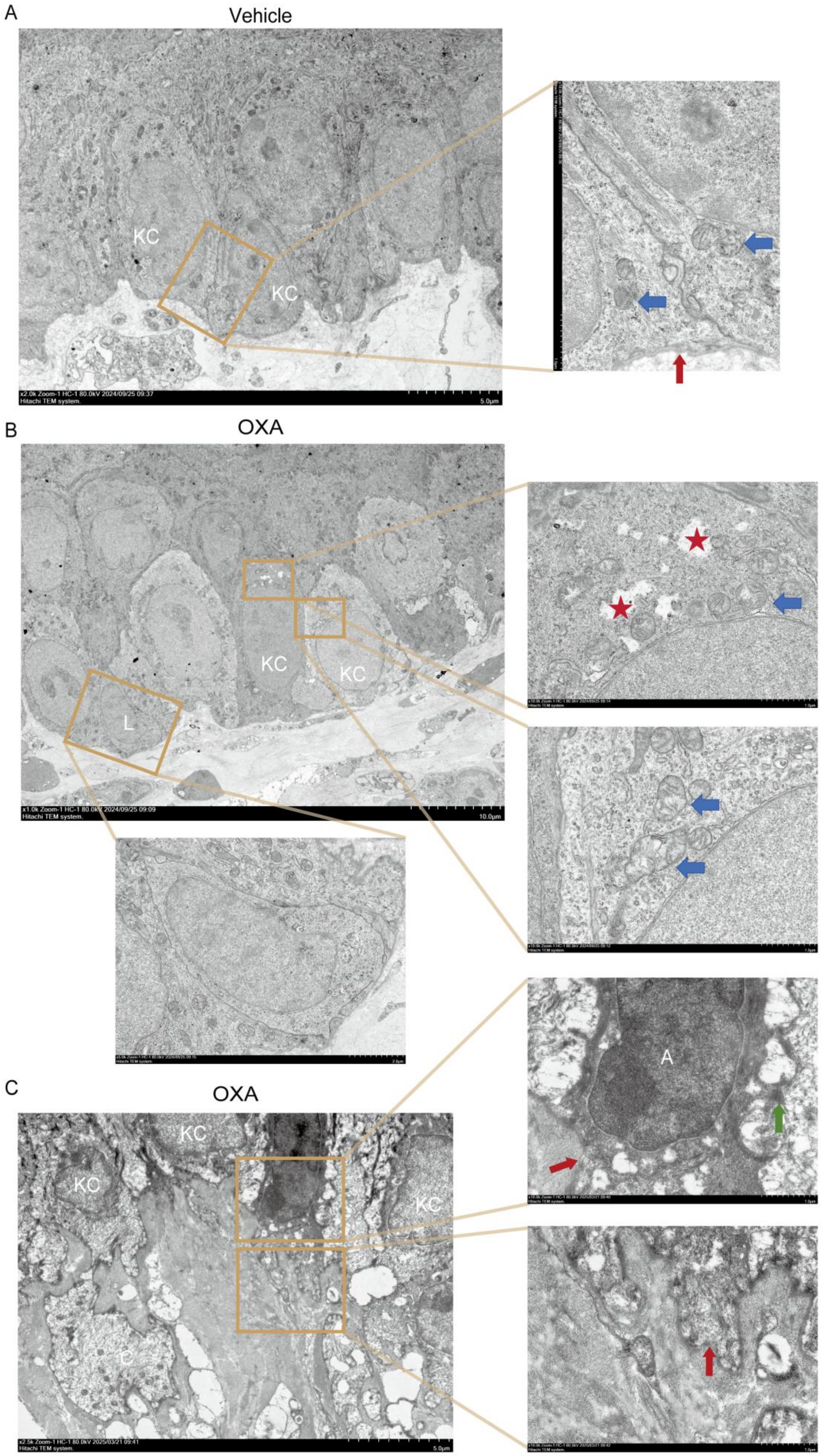
A
Vehicle
KC
KC
B
OXA
KC
KC
L
C
OXA
KC
KC
KC
C
A

Supplemental Figure 2. The ultrastructural changes around the basal membrane in the established oral lichen planus-like lesions

Transmission emission microscopy of labial mucosa from (A) vehicle (ethanol) or (B) OXA-treated mice. The low magnification image in the Vehicle group is at 2000× magnification, with a scale bar of 5 μm. The localized magnified image is at 10000× magnification, with a scale bar of 1 μm. In the OXA group, the low magnification image is at 1000× magnification, with a scale bar of 10 μm. The localized magnified image of lymphocytes is at 5000× magnification, with a scale bar of 2 μm. The localized magnified image showing intracellular edema and abnormal mitochondrial morphology is at 10000× magnification, with a scale bar of 1 μm. (C) altered basement membrane in the established oral lichen planus-like lesions. The low magnification image is at 2500× magnification, with a scale bar of 5 μm (the same image as shown in Figure 1D). The localized magnified images are at 10000× magnification, with a scale bar of 1 μm. Abbreviations: KC, keratinocyte; L, lymphocyte; Red star, intracellular edema; Blue arrow, mitochondria; Red arrow, basement membrane; Green arrow, desmosome.

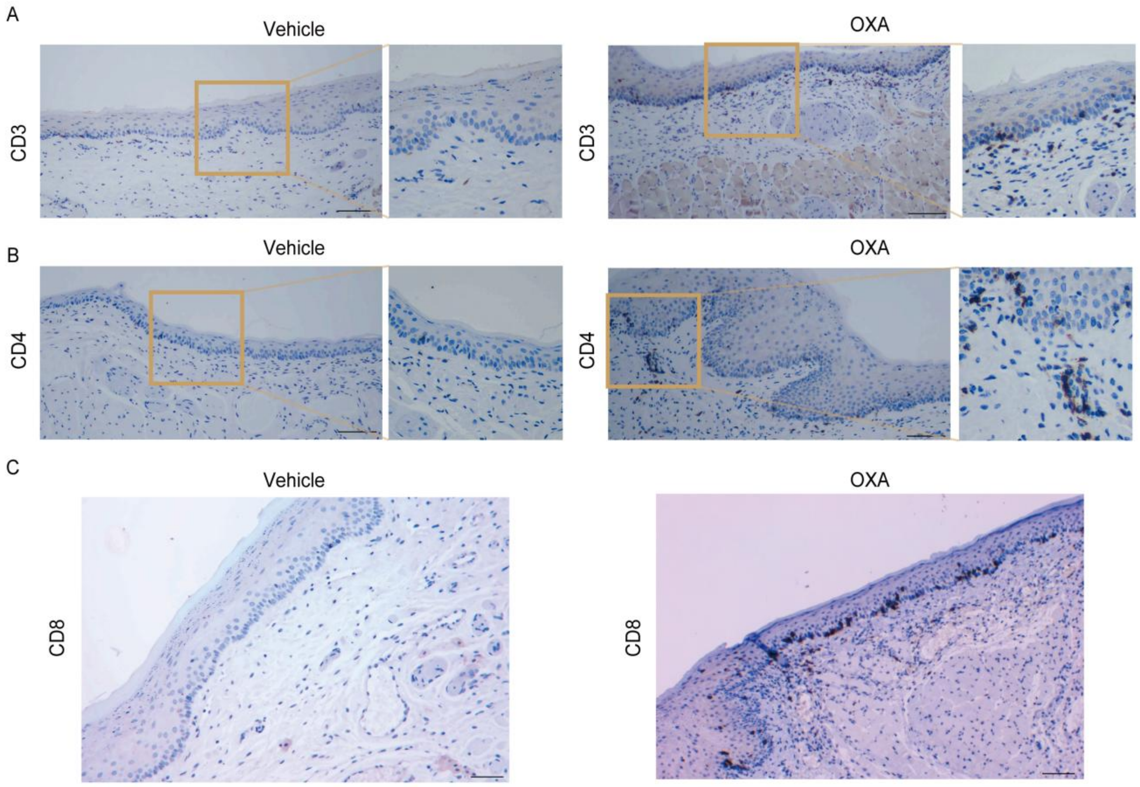

Supplemental Figure 3. Immunohistochemical images of CD3, CD4 and CD8 in oral lichen planus-like mouse model.

(A) Immunohistochemical images of vehicle or OXA-treated mice for CD3 (original magnification ×40 for low power field, scale bar = 100 μm; ×400 for high power field).

(B) Immunohistochemical images of vehicle or OXA-treated mice for CD4 (original magnification ×40 for low power field, scale bar = 100 μm; ×400 for high power field).

(C) Immunohistochemical images of vehicle or OXA-treated mice for CD8 (original magnification ×100, scale bar = 50 μm, high power field was shown in Figure 1F).

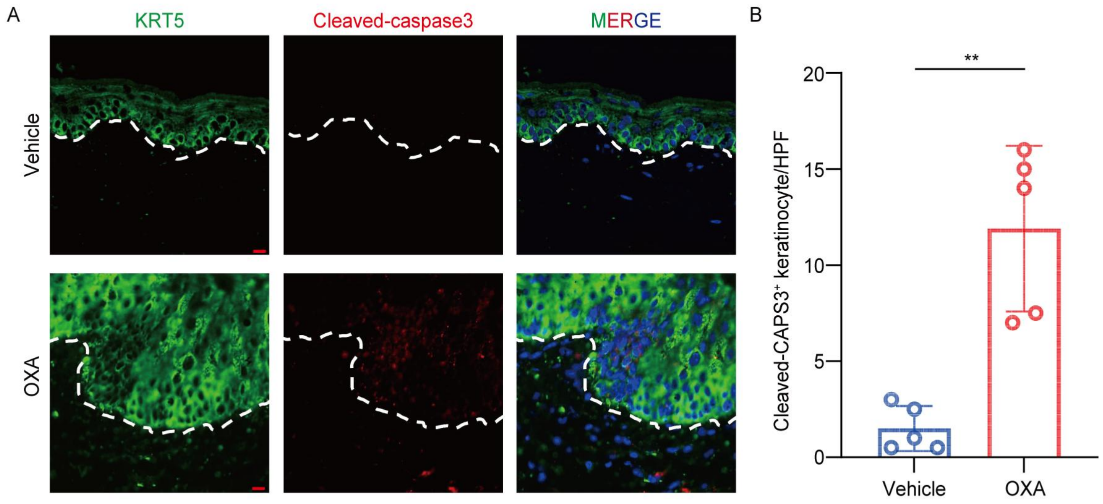

Supplemental Figure 4. Representative immunofluorescence images of cleaved caspase 3 and keratin 5

(A) Immunostaining of vehicle or OXA-treated mice for keratin5 (KRT5, green), cleaved caspase3 (red), and DAPI (blue; original magnification ×400, scale bar = 20 μm). White dash lines indicate epithelium-lamina propria junction. (B) Quantification of the cleaved-caspase 3^+ keratinocytes per high power field (HPF) in the vehicle or OXA-treated mice (n=5/group). Results are representative of three independent experiments. **$p < 0.01$. A two-tailed Student's *t*-test was used.

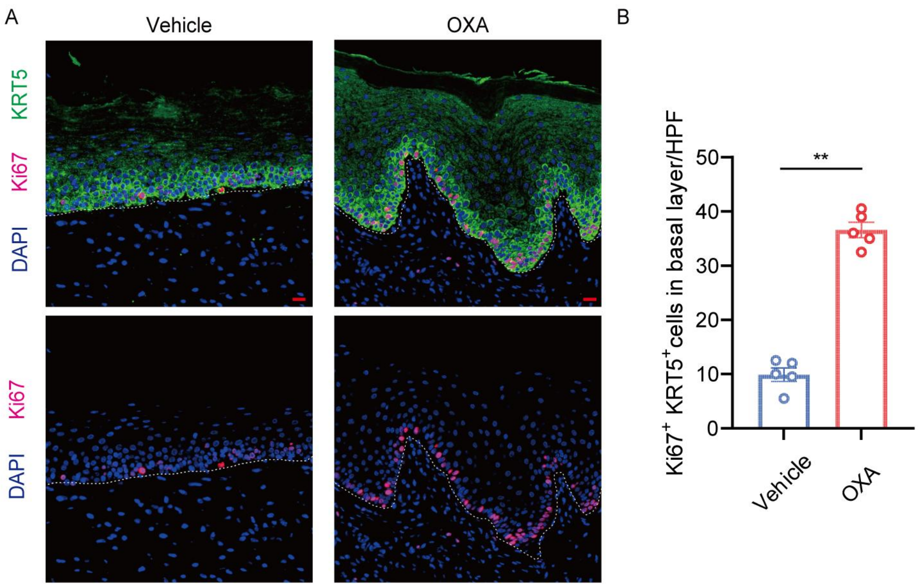

Supplemental Figure 6. Immunofluorescence co-staining of keratin 5 and Ki67 in normal mouse mucosa and oral lichen planus-like lesion sections.

(A) Immunostaining of vehicle or OXA-treated mice for Ki67 (violetgreen), keratin 5 (KRT5, green) and DAPI (blue; original magnification ×400, scale bar = 20 μm). (B) Quantification of the Ki67-positive KRT5^{+} cells in basal layer per high power field (HPF) in the vehicle or OXA-treated mice (n=5/group). Results are representative of three independent experiments. **p < 0.01. A two-tailed Student's *t*-test was used.

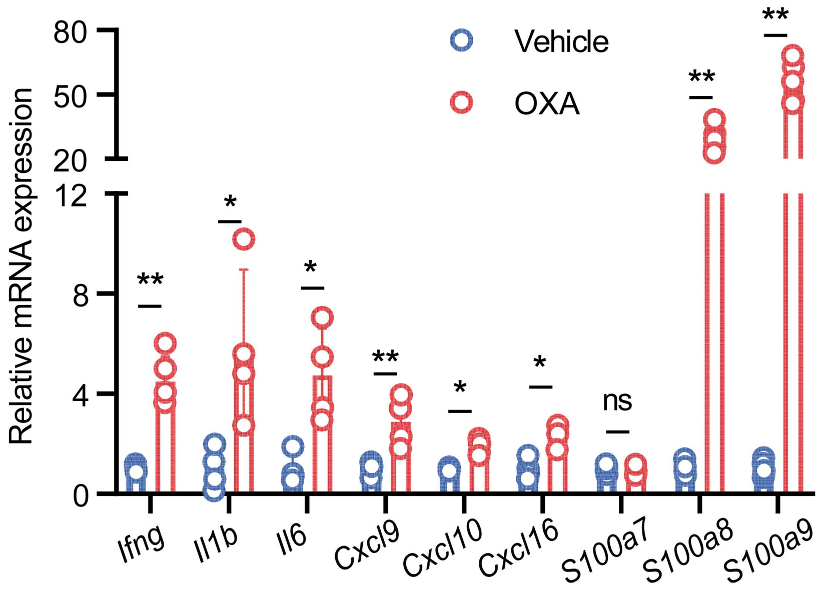

Supplemental Figure 7. Measurement of pro-inflammatory genes expression in oral lichen planus-like lesion by RT-qPCR

Relative mRNA expression levels of *Ifng*, *Il1b*, *Il6*, *Cxcl9*, *Ccxl10*, *Ccxl16, S100a7, S100a8* and *S100a9* were measured by RT-qPCR. Data are presented as mean ± SD (n = 4-5 per group). Results are representative of three independent experiments. Statistical significance between groups was determined by two-tailed Student's *t*-test (*p < 0.05, **p < 0.01, ns, not significant).

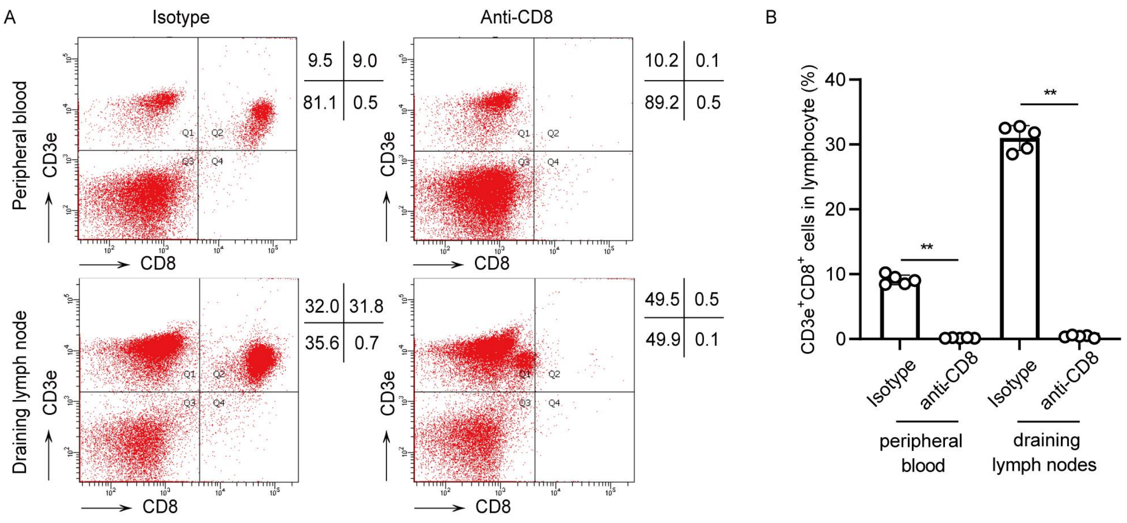

Supplemental Figure 8. Efficiency of CD8$^+$ T cell depletion in peripheral blood and draining lymph nodes assessed by flow cytometry.

One day after administering either isotype control or CD8-neutralizing antibodies to mice, single-cell suspensions were prepared from peripheral blood and draining lymph nodes (cervical). Flow cytometry analysis was performed by gating on the lymphocyte population in the FSC-SSC plot. (A) Dot plots compare the percentage of CD3e$^+$CD8$^+$ cells in peripheral blood and lymph nodes between the two groups. Results demonstrate significantly reduced proportions of CD8$^+$ T cells in the antibody-depletion group. (B) Quantification of the proportion of CD3e$^+$CD8$^+$ cells among lymphocytes in peripheral blood and draining lymph nodes. Each group consisted of five mice (n = 5). The results are representative of three independent experiments. A one-way ANOVA with a Tukey post hoc test was used.

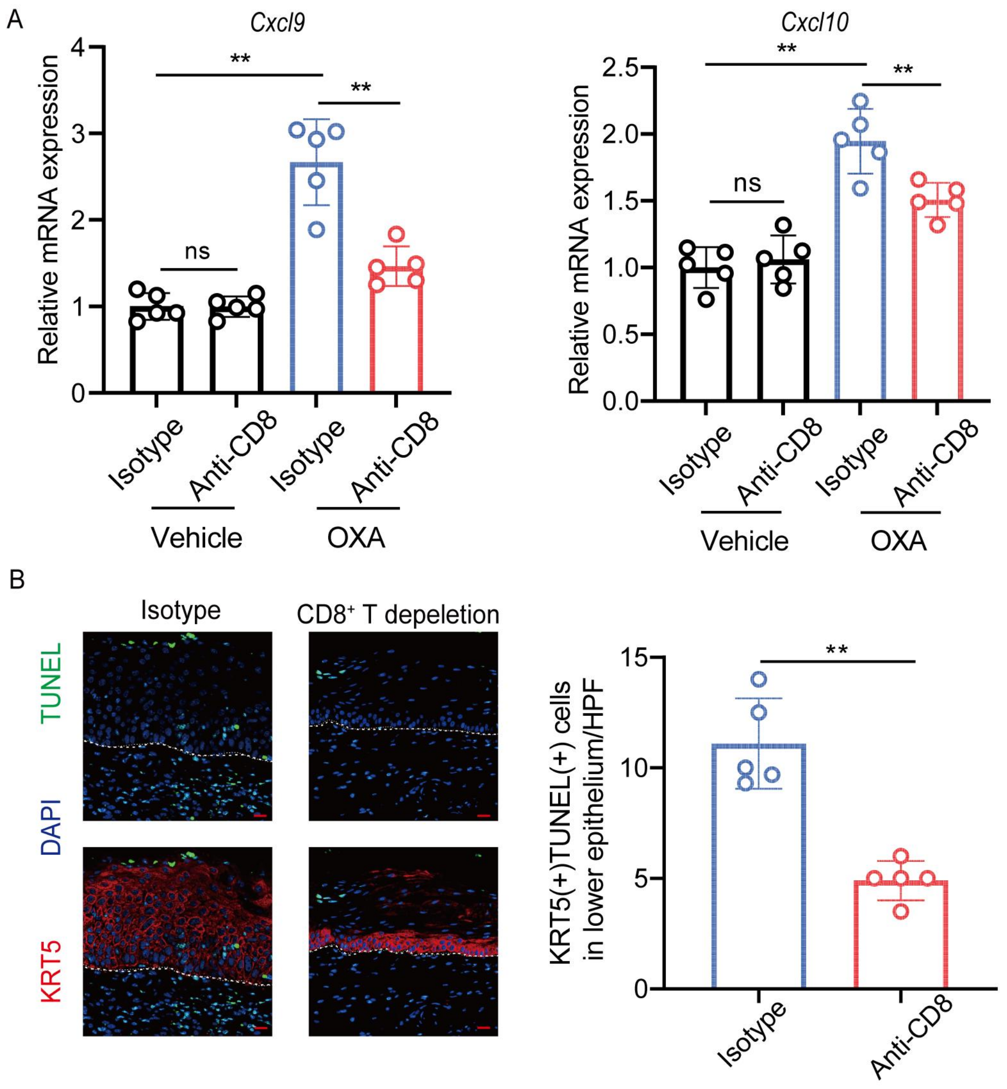

Supplemental Figure 9. Alterations in *Cxcl9* and *Cxcl10* expression and TUNEL^{+} keratinocytes following CD8 depletion in oral lichen planus-like lesions.

(A) Relative mRNA expression levels of *Cxcl9* and *Cxcl10* were measured by RT-qPCR (n= 5 each group). (B) Representative immunofluorescence images of keratin 5 (KRT5, red) and TUNEL (green) staining (original magnification ×200, scale bar = 50 μm). Quantification of KRT5^{+}TUNEL^{+} T cells in lower epithelium per high power field (HPF, n=5 each group). White dashed lines indicate the epithelium-lamina propria junction. Results are representative of three independent experiments. ns, not significant; **p < 0.01. A one-way ANOVA with a Tukey post hoc test was used for mRNA analysis. A two-tailed Student's *t*-test was used for KRT5(+)TUNEL(+) cells analysis.

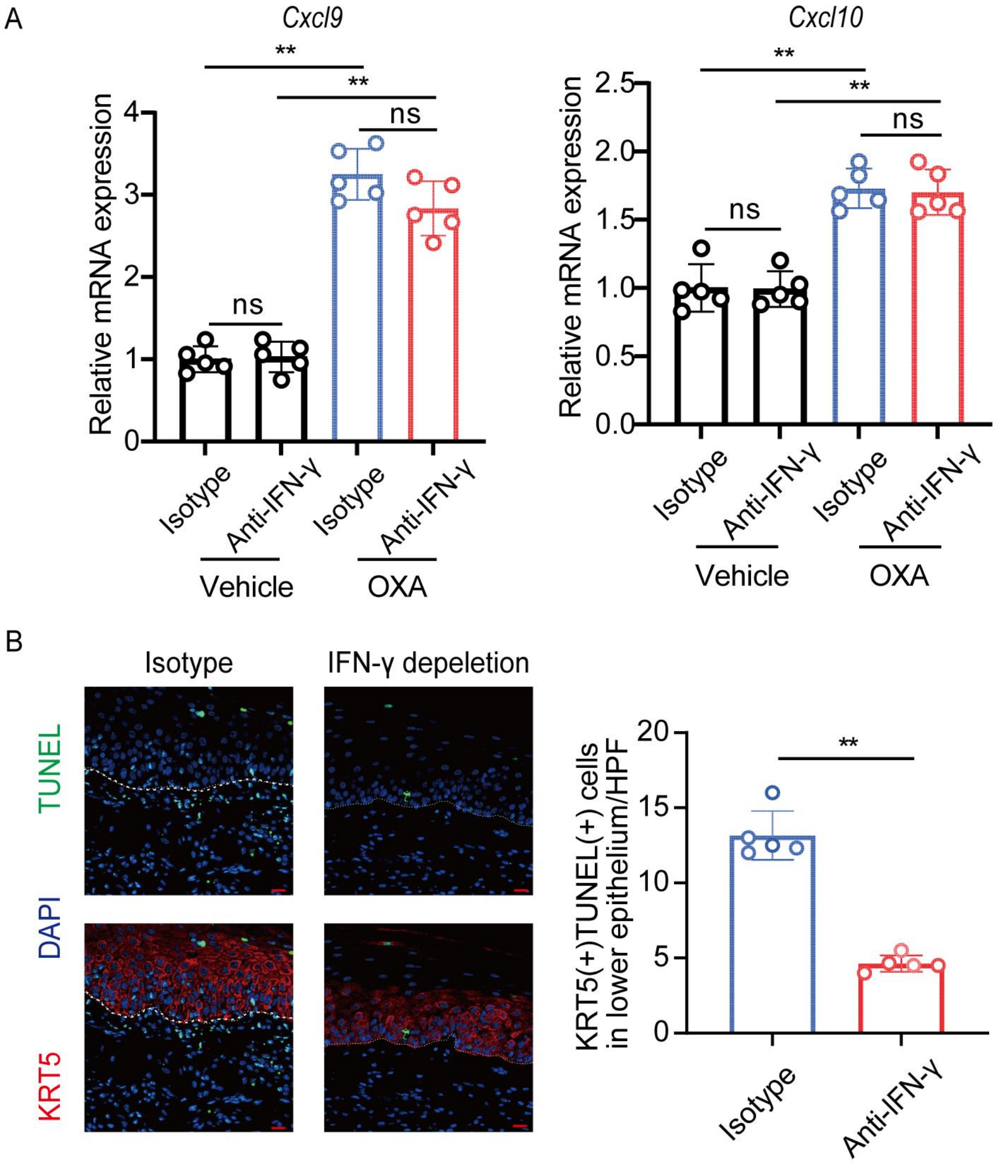

Supplemental Figure 10. *Cxcl9* and *Cxcl10* mRNA measurement and TUNEL$^+$ keratinocytes after IFN-γ depletion in oral lichen planus-like lesions.

(A) Relative mRNA expression levels of *Cxcl9* and *Cxcl10* were measured by RT-qPCR. (B) Representative immunofluorescence images of keratin 5 (KRT5, red) and TUNEL (green) staining (original magnification ×200, scale bar = 50 μm). Quantification of KRT5$^+$TUNEL$^+$ T cells in lower epithelium per high power field (HPF, n=5 each group). White dashed lines indicate the epithelium-lamina propria junction. Results are representative of two independent experiments. ns, not significant; **$p < 0.01$. A one-way ANOVA with a Tukey post hoc test was used for mRNA analysis. A two-tailed Student's

t-test was used for KRT5(+)TUNEL(+) cells analysis.

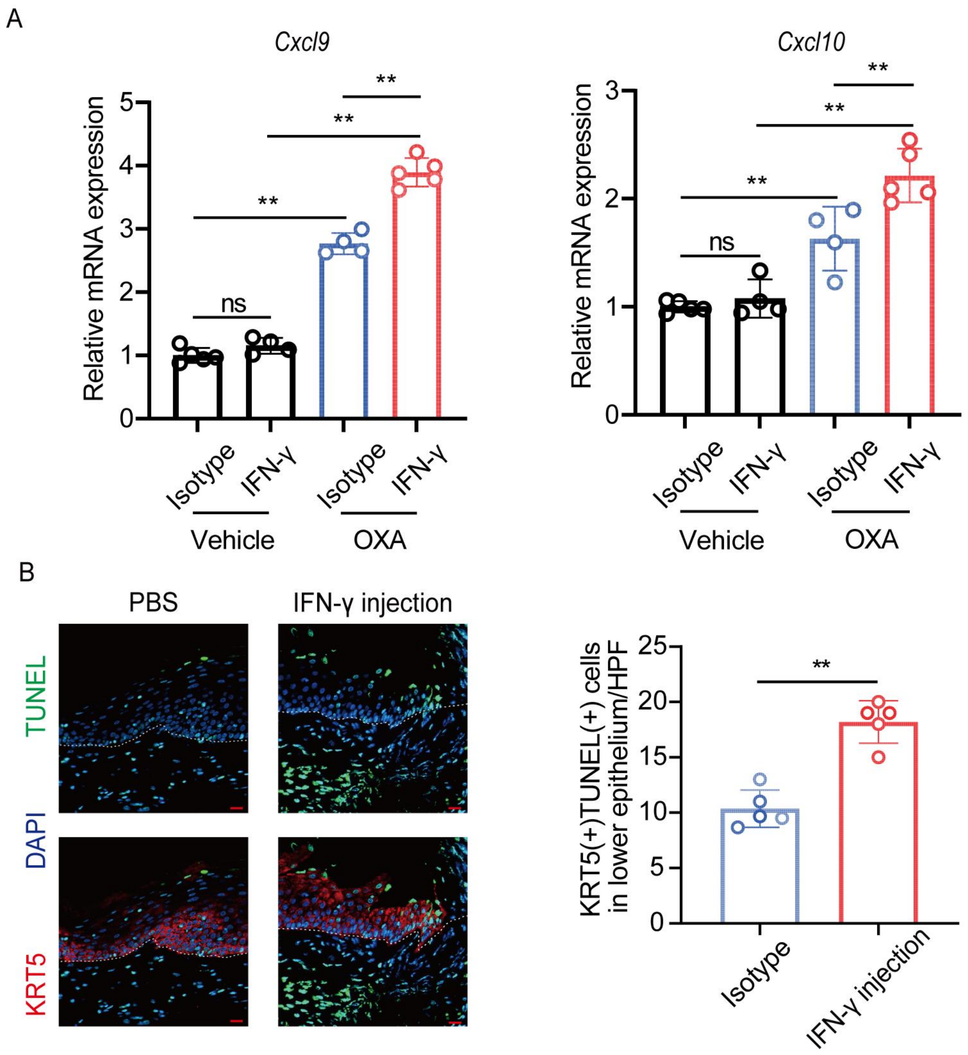

Supplemental Figure 11. Increased *Cxcl9*, C*xcl10* and TUNEL^{+} keratinocytes after IFN-γ injection in oral lichen planus-like lesions.

(A) Relative mRNA expression levels of *Cxcl9* and *Cxcl10* were measured by RT-qPCR. Data are presented as mean ± SD (n = 4-5 per group). (B) Representative immunofluorescence images of Keratin 5 (KRT5, red) and TUNEL (green) staining (original magnification ×200, scale bar = 50 μm). Quantification of KRT5^{+}TUNEL^{+} T cells in lower epithelium per high power field (HPF, n= 5 each group). White dashed lines indicate the epithelium-lamina propria junction. Results are representative of two independent experiments. ns, not significant; **p < 0.01. A one-way ANOVA with a Tukey post hoc test was used for mRNA analysis. A two-tailed Student's *t*-test was used for KRT5(+)TUNEL(+) cells analysis.

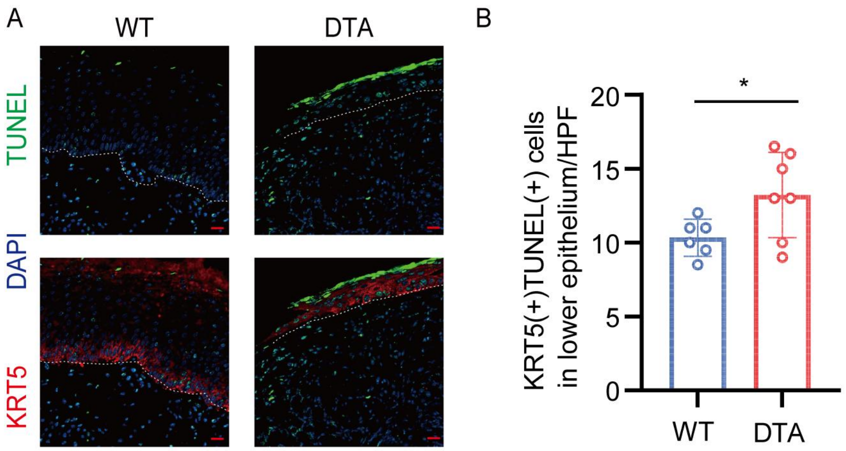

Supplemental Figure 12. Immunofluorescence staining of TUNEL^{+} cells in oral lichen planus-like lesions of wildtype and hLangerin-DTA mice.

(A) Representative immunofluorescence images of Keratin 5 (KRT5, red) and TUNEL (green) staining (original magnification ×200, scale bar = 50 μm). (B) Quantification of KRT5^{+}TUNEL^{+} T cells in lower epithelium per high power field (HPF, n=6-7 each group). Results are representative of two independent experiments. White dashed lines indicate the epithelium-lamina propria junction. ns, not significant. A two-tailed Student's *t*-test was used.

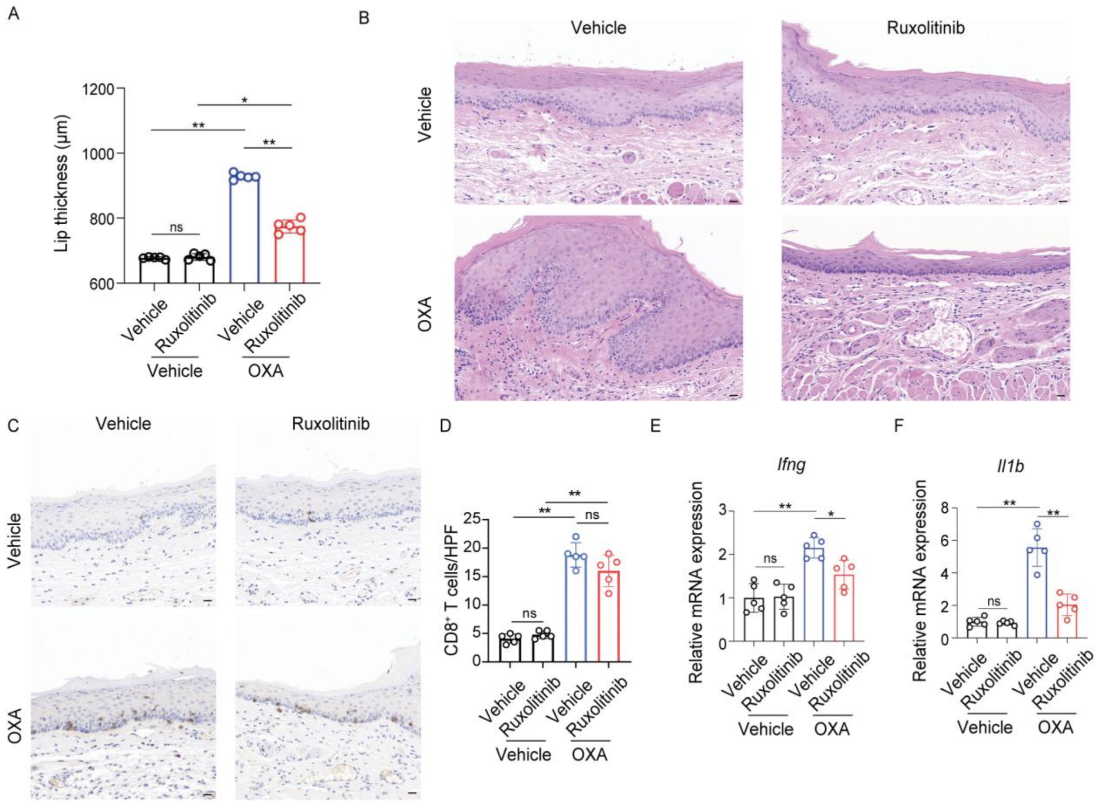

Supplemental Figure 13. Topical application of JAK inhibitor ruxolitinib ameliorates the oral lichen planus-like lesions.

For the therapeutic potential of topical JAK inhibitor, 1.5% ruxolitinib dissolved in DMSO were applied topically in the elicitation phase. (A) Lip thickness measurement on the fifth day. (B) Representative hematoxylin and eosin-stained sections from both groups (original magnification ×400, scale bar = 20 μm). (C) Representative immunohistochemical images of CD8 staining (original magnification ×400, scale bar = 20 μm). (D) Quantification of $CD8^+$ T cells per high power field (HPF). Relative mRNA expression of (E) *Ifng*, and (F) *Il1b* as determined by RT-qPCR. Data are presented as the mean ± SD (n = 5 mice for each group). Data represent one of two independent experiments. ns, not significant, *p < 0.05, ** p < 0.01. A one-way ANOVA with a Tukey post hoc test was used.

Supplemental Table 1. RT-qPCR primers for mRNA measurements.

Il1b	Forward	5’-GCCACCTTTTGACAGTGATG-3’
	Reverse	5’-AAGGTCCACGGGAAAGACAC-3’
Il6	Forward	5-TGCAAGAGACTTCCATCCAGT-3’
	Reverse	5-CTGCAAGTGCATCATCGTTGT-3’
Ifng	Forward	5-ACTGGCAAAAGGATGGTGAC-3’
	Reverse	5-ACCTGTGGGTTGTTGACCTC-3’
H2-d1	Forward	5’-TGGGGTCATAACCCTCACCTT-3’
	Reverse	5’-GAAAACTCAGACCCTGCCCT-3’
H2-q4	Forward	5-ATGGCGTCAACAATGCTGC-3’
	Reverse	5-GGGACACGGAGGTGTAGAA-3’
Cxcl9	Forward	5’-GGAGTTCGAGGAACCCTAGTG-3’
	Reverse	5’-GGGATTTGTAGTGGATCGTGC-3’
Cxcl10	Forward	5'-GCCGTCATTTTCTGCCTCAT-3’
	Reverse	5'-GCTTCCCTATGGCCCTCATT-3’
Cxcl16	Forward	5’-CCTTGTCTCTTGCGTTCTTCC-3’
	Reverse	5’-TCCAAAGTACCCTGCGGTATC-3’
S100a7	Forward	5’-TCCATGATAGTGGCCTCTCAGA-3’
	Reverse	5’-GGGTCAATCCTTGGTCCATCTT-3’
S100a8	Forward	5’-TCAGCTCCGTCTTCAAGACATC-3’
	Reverse	5’-TGTAGAGGGCATGGTGATTTCC-3’
S100a9	Forward	5’-ATACTCTAGGAAGGAAGGACACC-3’
	Reverse	5’-TCCATGATGTCATTTATGAGGGC-3’
Hprt	Forward	5’-TGGATACAGGCCAGACTTTG-3’
	Reverse	5’-GATTCAACTTGCGCTCATCTTA-3’
